# Linezolid induced hypoglycemia and anemia: A case report

**DOI:** 10.1002/ccr3.7713

**Published:** 2023-07-30

**Authors:** Xiao‐hua Lan, Yue Hu, Yan‐jie Cao, Ping Liu, Qi‐tao Ren, Wei‐wei Zhu

**Affiliations:** ^1^ Air Force Medical Center, PLA Beijing China; ^2^ Graduate School of Hebei North University Zhangjiakou Hebei Province China; ^3^ Graduate School of China Medical University Shenyang Liaoning Province China

**Keywords:** adverse reactions, anemia, case reported, hypoglycemia, linezolid

## Abstract

**Key Clinical Message:**

Linezolid (LZD) is an efficient addition antibiotic against multidrug‐resistant strains. However, clinicians should pay attention to the adverse reactions such as hypoglycemia and anemia in using LZD, especially in elderly patients and patients with abnormal liver and kidney function who need to use LZD for a long time.

**Abstract:**

Severe hypoglycemia and anemia caused by linezolid (LZD) are rare, with potentially serious adverse effects. The report of LZD‐induced hypoglycemia and anemia is extremely rare. Thus far, this is the first report. We presented LZD‐induced recurrent hypoglycemia and anemia in a 93‐year‐old patient who has been prescribed LZD 600 mg once daily for 42 days for treatment of tuberculosis (TB) pleurisy and pneumonia. The patient began to experience recurrent hypoglycemic episodes and anemia 5 days and 2 weeks after LZD medication, respectively. Using Naranjo's Adverse Drug Reaction Assessment Scale, the patient scored 8 points with the category of “probable”. His hypoglycemia and anemia gradually improved 1 month after LZD withdrawal. Clinicians should pay attention to the adverse reactions such as hypoglycemia and anemia in using LZD, especially in elderly patients and patients with abnormal liver and kidney function who need to use LZD for a long time. Patients should regularly monitor blood routine, blood glucose, and liver and kidney functions during LZD exposure, which may avoid adverse reactions and improve their prognosis.

## Graphical abstract

Change in Hb during LZD therapy and withdrawal.

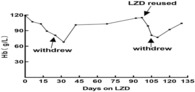

## 1 INTRODUCTION

Linezolid (LZD) is a synthetic antibiotic which block the synthesis of bacterial protein through binding to rRNA on both the 30S and 50S ribosomal subunits.^1^ LZD has been reported in clinical studies as an efficient addition antibiotic against multidrug‐resistant strains, such as methicillin‐resistant *S. aureus* (MRSA), vancomycin‐resistant enterococcus faecium (VREF), multidrug‐resistant tuberculosis (MDR‐TB) or extensively drug‐resistant TB (XDR‐TB). It is also utilized by the ICU patients to treat Gram‐positive infections commonly caused by multidrug‐resistant strains including MRSA and VREF. Plasma concentrations of LZD in elderly patients, patients with mild‐to‐moderate hepatic damage, or patients with mild‐to‐chronic renal failure are similar to those in younger or healthy volunteers,^2^ but there are still studies that suggest dose reduction may improve safety outcomes among patients with renal impairment.^3^ LZD has relatively mild and temporary side effects including neuropathy, reversible myelosuppression, hyperlactatemia, diarrhea, nausea, headache, and hypoglycemia. Severe hypoglycemia and anemia caused by LZD are rare and have potentially serious adverse effects. To our knowledge, there are no reports of LZD‐induced hypoglycemia and anemia. We report a case of reversible hypoglycemia and anemia after 42 days of LZD treatment.

## 2 CASE REPORT

A 93‐year‐old man was admitted to the hospital with diagnosis of TB pleurisy on March 17, 2021. He had a history of diabetes mellitus type 2 (T2DM), hypertension, chronic kidney disease, chronic lung inflammation, etc. Physical examination on admission: left lung breath sounds lower, and a small amount of fine wet rales in both lungs. Laboratory examinations: fasting blood glucose: 11.6 mmol/L, white blood cell (WBC): 7.87 × 10^9^/L, C‐reactive protein (C‐RP): 14 mg/L; PCT: 0.086 ng/mL; red blood cell (RBC): 3.76 × 10^12^/L, hemoglobin: 119 g/L, serum creatinine(Scr): 84 umol/L, brain natriuretic peptide (BNP): 105.9 pg/mL; negative for occult blood in stool; positive for mycobacterium tuberculosis antibody test. X‐ray chest: chronic interstitial inflammation of both lungs; left pleural effusion. Pleurisy caused by other causes, such as tumor, cardiac and renal insufficiency, endocrine and metabolic diseases, and malnutrition, were almost excluded. The diagnosis of TB pleurisy could not be excluded. Therefore, he was treated with LZD 600 mg/day, isoniazid and Levofloxacin tablets. He began to experience restlessness, sweating, and limb convulsions with blood sugar at 2.6 mmol/L level on the 5th day of treatment with LZD. The symptoms improved by nasal feeding of 50% GS. After that, the dose of insulin aspart was reduced according to the blood glucose level. On eighth day of treatment with LZD, the symptoms of nausea, profuse sweating, and restlessness recurred with blood glucose at 2.5 mmol/L and improved gradually after nasal feeding with 50% GS. On the 19th day of treatment with LZD, the blood routine indicated: WBC: 8.81 × 10^9^/L, CRP: 8 mg/L, RBC: 2.8 × 10^9^/L, Hb: 89 g/L, hematocrit (HCT): 0.26 L/L; fecal occult blood test negative. LZD had to be withdrawn in the fourth week of treatment as a result of hypoglycemia and Hb decline again. Blood test on sixth day of LZD withdrawal showed RBC 2.24 × 10^9^/L, Hb 68 g/L, HCT 0.21 L/L; on third week of LZD withdrawal: WBC 8.57 × 10^9^/L, CRP 14 mg/L, RBC 3.08 × 10^9^/L, Hb 103 g/L. No hypoglycemic‐related symptoms recurred since LZD withdrawal.

**FIGURE 1:**
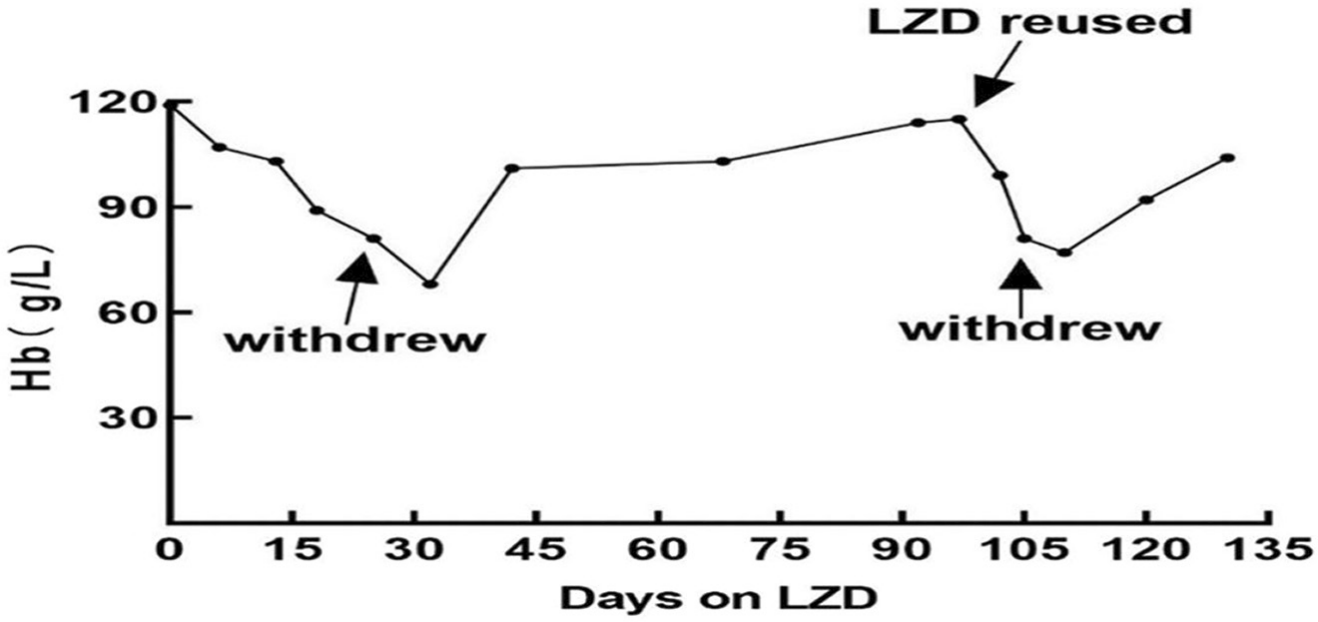
Change in Hb during linezolid (LZD) therapy and withdrawal.

The patient developed pneumonia on June 17, 2021 with WBC: 12.46 × 10^9^/L, RBC: 3.49 × 10^12^/L, Hb: 114 g/L, CRP: 31.23 mg/L, fasting blood glucose: 11.5 mmol/L. LZD was given intravenously 0.6 g every 12 h combined with imipenem according to sputum culture results. The symptoms of restlessness, sweating, and twitching of the limbs recurred after 4 days of this treatment with blood sugar at 2.5 mmol/L level. After 2 weeks, the laboratory examination indicated WBC: 7.48 × 10^9^/L, RBC: 2.54 × 10^12^/L, HB: 81 g/L, CRP: 8.74 mg/L, PCT: 0.241 ng/mL; fecal occult blood negative. LZD had to be withdrawn because of repeated hypoglycemia and aggravation of anemia. Laboratory examination 2 weeks after LZD withdrawal indicated WBC: 7.21 × 10^9^/L, RBC: 2.87 × 10^12^/L, HB: 92 g/L; CRP: 8.12 mg/L; blood sugar levels increased gradually. Values of Hb varied during LZD use and withdrawal showed in Figure 1. LZD had not been used in this patient since then.

## 3 DISCUSSION

In recent years, it is not uncommon to report cases of hypoglycemia or anemia caused by LZD. Previous studies^4^, ^5^, ^6^ have shown that long‐term repeated use of LZD can lead to adverse reactions such as hypoglycemia, pernicious anemia, pancreatitis, and lactic acidosis. There are few reports of LZD‐induced hypoglycemia and anemia. To our knowledge, we are the first to report hypoglycemia and a drop in hemoglobin and red blood cells in patients on LZD therapy. It scored 8 points shown in Table 1 and rated as “probable” level according to Naranjo's Adverse Drug Reaction (ADR) Assessment Scale.^7^

**TABLE 1:** Adverse drug reaction (ADR) probability scale.

Parameters	Yes	No	Do not know	Respond	Score
1. Are there previous conclusive reports on this reaction?	✓			Yes	+1
2. Did the adverse event appear after the suspected drug was administered?	✓			Yes	+2
3. Did the adverse reaction improve when the drug was discontinued or a specific antagonist was administered?	✓			Yes	+1
4. Did the adverse reaction reappear when the drug was readministered?	✓			Yes	+2
5. Are there alternative causes (other than the drug) that could on their own have caused the reaction?		✓		No	+2
6. Did the reaction reappear when a placebo was given?			✓	Do not know	0
7. Was the drug detected in the blood(or other fluids) in concentrations known to be toxic?			✓	Do not know	0
8. Was the reaction more severe when the dose was increased, or less severe when the dose was decreased?			✓	Do not know	0
9. Did the patient have a similar reaction to the same or similar drugs in any previous exposure?		✓		No	0
10. Was the adverse event confirmed by any objective evidence?		✓		No	1
In total					8

Abbreviation: ADR, adverse drug reaction.

The mechanisms underlying LZD‐induced hypoglycemia are not clear, but this mechanism is considered to be related to the inhibition of monoamine oxidase, and the action of hydrazine‐type monoamine oxidase is important.^8^ In addition to its antibacterial effect, LZD also has the pharmacological activity of monoamine oxidase inhibitor (MAOI), which can prevent the normal metabolism of neurotransmitters norepinephrine, dopamine and 5‐hydroxytryptamine. MAOI has the characteristics of promoting insulin secretion and increasing insulin sensitivity.^9^, ^10^ Therefore, the occurrence of hypoglycemia would be a logical side effect of LZD therapy. A study^8^ showed that the median time of LZD‐induced hypoglycemia initiation was 7 days, and the median blood glucose lowest point was 1.77 mmol/L, which was basically consistent with our study. In addition, we found an interesting phenomenon that patient experienced hypoglycemia several days after discontinuation of LZD, which could not be prevented by adjusting the hypoglycemic regimen until after discontinuation of LZD. The hypoglycemia profile of our patient was very similar to the study by Viswanathan et al.,^11^ who reports on a 64‐year‐old diabetic patient taking LZD‐induced hypoglycemia for cellulitis. Previous studies have confirmed that hypoglycemia can occur in both diabetes and non‐diabetes patients. Our patient is a diabetic, but whether diabetic patients have a greater risk of hypoglycemia may be one of the future research directions. Although the mechanism of hypoglycemia in LZD is still unclear, the latest instructions of LZD suggest that clinicians should be alert to the occurrence of saccharification events when using LZD for diabetes patients.

Reversible myelosuppression following LZD treatment have been reported in phase 3 clinical trials of LZD and other recent studies, and the mechanism is similar to chloramphenicol‐induced myelosuppression,^12^ which inhibits protein synthesis in human mitochondria. Our patient developed anemia, and previous studies have shown that mitochondrial dysfunction caused by long‐term LZD application may be related to LZD‐induced PRCA protoxicity.^13^ Studies suggest that linezolid can inhibit mitochondrial respiration by breaking down mitochondrial protein synthesis and inhibiting protein synthesis by blocking the formation of the initiation complex^14^; therefore, LZD can lead to lactic acidosis, myelosuppression, pancreatitis, and other adverse consequences.

The multivariate logistic regression model of LZD‐induced anemia by Dai Ying et al.^15^ showed that patients with moderate to severe renal insufficiency and patients with a trough concentration of ≥7 mg/L had an increased risk of anemia, and patients receiving high initial doses of LZD are more likely to experience myelosuppression.^16^ However, the instructions of LZD indicate that the pharmacokinetic parameters of patients with hepatic and renal insufficiency will not change significantly, and there is no need to adjust the dose according to the hepatic and renal function, but we believe that this controversy needs to be further clarified in clinical practice. In this study, our patient developed hemoglobin and erythrocytopenia. Nine cases^17^, ^18^, ^19^, ^20^, ^21^, ^22^, ^23^ had previously reported that LZD induced anemia in linezolid, and the study found that anemia was reversible after drug discontinuation. It may be because LZD relieves the inhibition of mitochondrial protein synthesis. Our patient is an elderly patient. Studies by Yan Qin et al.^12^ confirmed that 62.5% of the patients who developed anemia after using linezolid were ≥ 60 years old, and the proportion of male patients was relatively large. The basic characteristics of our patients are consistent with the above studies. The patients we reported developed a decrease in Hb and RBC after 2 weeks of LZD exposure. We withdrew from LZD and gave corresponding treatment. After 4 weeks, the anemia returned to normal. This feature is basically consistent with the previous research by Yan Qin et al.^12^ that the recovery time of anemia caused by LZD induced 27 days. This indicates that the anemia caused by LZD is time‐dependent and dose‐dependent, which may be the result of a gradual increase in plasma concentrations in the later stages of treatment. In addition, for the treatment of anemia caused by LZD, relevant studies have shown^21^, ^23^ that supplementation of iron, vitamins, folic acid, and EPO cannot prevent and delay the occurrence of anemia, so regular monitoring of related anemia indicators appears to be more important.

In conclusion, clinicians should pay attention to the adverse reactions such as hypoglycemia and anemia in the process of using LZD, especially in elderly patients and patients with abnormal liver and kidney function who need to use linezolid repeatedly for a long period of time. It is recommended to use linezolid regular monitoring of blood biochemical routines and drug concentrations during the course of treatment to reduce adverse events and enhance clinical drug safety.

## AUTHOR CONTRIBUTIONS

**Xiao‐hua Lan:** Methodology; writing – original draft. **Yue Hu:** Formal analysis; writing – original draft. **Yan‐jie Cao:** Funding acquisition; methodology; resources; writing – original draft. **Ping Liu:** Formal analysis; investigation. **Qi‐tao Ren:** Investigation. **Wei‐wei Zhu:** Investigation.

## FUNDING INFORMATION

This research was funded by Teaching Research Project of Air Force Medical Center (2021JX001).

## CONFLICT OF INTEREST STATEMENT

The authors declare that they have no competing interests.

## ETHICS APPROVAL AND CONSENT TO PARTICIPATE

Not applicable.

## CONSENT

Written informed consent for publication of the clinical details and clinical images was obtained from the patient's guardian.

## Data Availability

All data generated or analyzed in the present study are included in this published article.
